# Supplementary material for: Two decades of change in sea star abundance at a subtidal site in Puget Sound, Washington
Source: PLoS One. 2023 Jun 9;18(6):e0286384. doi: 10.1371/journal.pone.0286384 (PMC10256211; doi:10.1371/journal.pone.0286384)
Supplement: Attachment — Submitted filename: Review of Casendino et al Plos One_Carr.docx [file pone.0286384.s001.docx]

Review of Casendino – PONE-D-22-2571

“Two decades of change in sea star abundance at a subtidal site in Puget Sound, Washington”

**General comments:** This study benefits from three key aspects. First, as the author’s point out, it leverages a rare and fortunate long-term timeseries. Secondly, the analyses are excellent. Third, it is an impressive use of a dataset generated from a field sampling project for a course. However, the study suffers from writing that doesn’t provide a strong conceptual motivation, and insufficient depth and breadth to provide a convincing story.

Taken on face value (i.e. trends in all species combined), the analyses strongly suggest that factors other than SSWD are responsible for the trajectories of sea stars across depths over time. If the authors quantitatively evaluated these alternative explanations and found stronger relationships, this argument would not only be more compelling, but also be the basis of an interesting story, which is exactly how they close the Discussion section. This could have been the overarching theme of the article, “Contrary to other regions of the West Coast-wide SSWD epidemic, other potential environmental drivers (potential because they are all correlative), overwhelm the relative impact of SSWD in an assemblage of sea stars in Puget Sound.” That is a very interesting results, but requires more analyses.

The other problem I have with the manuscript is the lack of information (temporal trends by depth) for each species. Yes, they are insufficient for rigorous statistical analysis, but not knowing how the different species are potentially contributing to trends is extremely frustrating. The authors are not providing information at the species level that readers would find intriguing.

Overall, because the manuscript describes a very localized phenomenon with little broader conceptual contributions, I think the manuscript is more appropriate for a more regional or disciplinary-focused journal. A marine or environmental journal whose readers would appreciate that wide-scale manifestations of a disease were overwhelmed locally by environmental degradation is something I’d be keen to cite.

**Specific comments:**

**Introduction**

I deeply appreciate the emphasis on the value of long-term data sets, but it is not the point of the article. Start the Intro focused on the greater conceptual issue. If the overall conclusion is how local environmental conditions can overwhelm the effects of otherwise disastrous widescale perturbations like epidemics, start with that and how this knowledge advances our understanding of the causes of spatial variation in such perturbations.

Certainly include the point of how long term datasets are central to addressing this issue and also consider additional citations. Especially look for the most recent articles by Lindenmayer:

- Hughes, B.B., Beas-Luna, R., Barner, A.K., Brewitt, K., Brumbaugh, D.R., Cerny-Chipman, E.B., Close, S.L., Coblentz, K.E., De Nesnera, K.L., Drobnitch, S.T. and Figurski, J.D., 2017. Long-term studies contribute disproportionately to ecology and policy. *BioScience*, *67*(3), pp.271-281.
- Lindenmayer DB, Likens GE, Krebs CJ, Hobbs RJ. 2010. Improved probability of detection of ecological “surprises.” Proceedings of the National Academy of Sciences 107: 21957–21962.
- Lindenmayer DB, et al. 2012. Value of long-term ecological studies. Austral Ecology 37: 745–757.

The purpose of the paragraphs describing the significance of the SSWD should be terse and describe the timing, geographic scope and species impacted, pointing out that it occurred in the intertidal and subtidal. Point made and no greater detail is necessary. There are several articles that describe this phenomenon for subtidal (especially, *P. helanthoides*) ecosystems, even though they also draw attention to the role of the MHW. They should be cited as they contribute to geographic scope and ecological consequences associated, in part, to SSWD.

- McPherson, M.L., Finger, D.J., Houskeeper, H.F., Bell, T.W., Carr, M.H., Rogers-Bennett, L. and Kudela, R.M., 2021. Large-scale shift in the structure of a kelp forest ecosystem co-occurs with an epizootic and marine heatwave. *Communications biology*, *4*(1), pp.1-9.
- Smith, J.G., Tomoleoni, J., Staedler, M., Lyon, S., Fujii, J. and Tinker, M.T., 2021. Behavioral responses across a mosaic of ecosystem states restructure a sea otter–urchin trophic cascade. *Proceedings of the National Academy of Sciences*, *118*(11), p.e2012493118.

Line 77- “Eisenlord et al. (2016) tracked 78 SSWD prevalence in *P. ochraceus* in Washington State from December 2013 to July 2015 [17].” Summarize this entire study in 1-2 sentences as one of several examples of spatial variation in the population consequence of SSWD, which is the key point of this article (i.e. that it was not manifest strongly at the study site).

**Methods**

Line 139; “Each year, five trawls took place at each depth, 140 corresponding to discrete time periods: early morning (~6:00–8:00), morning (~10:00–12:00), 141 afternoon (~15:00–17:00), evening (~20:00–22:00), and night (~1:00–3:00). Each trawl sampled 142 benthic habitat for ~5 min over 370 m (range = 367 to 538 m).”

Please make clearer the spatial distribution of transects. Was essentially the same transect area sampled repeatedly over time on a sampling day, or was the location of the transects at a given depth stratum offset so as not to sample the very same area over time? We’re you depleting the stars over the sequence of the samples and summing them across samples? Did catch at each depth decline over the day?

**Results**

**Table 2:** “listed in order of overall abundance”. To make this more obvious and informative, add a column for mean abundance across all depth strata for each species.

Line 299: “The presence of relatively resilient *D. imbricata* in shallow subtidal depths alone may also have offset localized mortality rates, perhaps explaining why high abundances of *P. helianthoides* (and SSWD-associated mortality) in the shallow subtidal were not associated with more drastic declines in those depths. Unfortunately, replication at the level of species was insufficient to test for species-specific trends in our dataset.”

Yes, you can’t statistically evaluate change for each species by depth, but not seeing graphically how each species changed at each depth doesn’t allow the reader (or you) to evaluate the potential contributions of each species to the trends of combined species and that is frustrating. You need to make supplemental graphs of the trends in density for each species by each depth stratum. As the authors indicate, *P. helianthoides* might have tanked at shallower depths but it is masked by lack of change in *D. imbricata.* Readers need to see the trends for each species, as poor as they are because of such low densities, to interpret the trends in the combined species.

Line 325: “In the context of these reports, our 326 results strongly suggest a lagged onset of SSWD-associated mortality at Port Madison.” But again, it is frustrating not to know which species contributed most to this pattern. What if the major contributors were species thought not to be susceptible to SSWD?

Line 335: “… suggesting that declines may have resulted from a deleterious environmental variable. Since 1999, the frequency of phytoplankton blooms and average chlorophyll-a in Puget Sound have declined;…”

An analysis of how well these other environmental variables explained the observed population trend would be very interesting. The take home of such a study would be “Yeah, SSWD was devastating in many parts of the West Coast, but environmental degradation (or natural long-term trends) in Puget Sound overwhelmed such effects!”. That’s interesting!

Line 343: “we expected high temperatures to be correlated with low sea star density…” Yes, but your points as to why higher temps could be detrimental (e.g.,. hypoxia) beg the question of whether any species were migrating across depth zones either to avoid detrimental conditions or in pursuit of more beneficial conditions (prey availability). How species number changed across depth zones over time would be interesting and might explain these surprising relationships.

Line 367: This work suggests that subtidal sea star communities experienced gradual and steep

declines not strictly attributable to the SSWD epizootic of 2013, warranting further examination

of other external threats posed to such communities in the Salish Sea.” That says it all, and should be the title and point of this article.
